# Effect of an Experimental Formulation Containing Chlorhexidine on Pathogenic Biofilms and Drug Release Behavior in the Presence or Absence of Bacteria

**DOI:** 10.3390/pharmaceutics11020088

**Published:** 2019-02-19

**Authors:** Ana Carolina S. Ré, Maria Carolina Bonjovanni, Maíra P. Ferreira, Osvaldo Freitas, Carolina P. Aires

**Affiliations:** 1 Department of Physics and Chemistry, School of Pharmaceutical Sciences of Ribeirão Preto, University of São Paulo, Ribeirão Preto, CEP 14040-903, São Paulo, Brazil; ana3.santos@usp.br (A.C.S.R.); carolinabonjovanni@hotmail.com (M.C.B.); 2 Department of Pharmaceutical Sciences, School of Pharmaceutical Sciences of Ribeirão Preto, University of São Paulo, Ribeirão Preto, CEP 14040-903, São Paulo, Brazil; maira@fcfrp.usp.br (M.P.F.); ofreitas@fcfrp.usp.br (O.F.)

**Keywords:** chlorhexidine, oral cavity, semi-solid systems, drug quantification, biofilms

## Abstract

(1) Background: For any antibacterial oral formulation to be successful, it must present effects in the presence of biofilms. Therefore, our aim is to analyze the drug release and the antibiofilm effects of a semi-solid formulation containing chlorhexidine (CHX) in the presence of pathogenic biofilms. (2) Methods: The biofilms of *Streptococcus mutans* (*n* = 6) or *Porphyromonas gingivalis* (*n* = 3) were formed for 6 and 4 days, respectively, being exposed to: 1) a CHX system or 2) vehicle control without CHX. A group without treatment was included as negative control. The acidogenicity, CHX quantification and bacterial viability were determined. A dissolution assay in a buffer and culture medium in the absence of bacteria was also performed. (3) Results: Although the CHX quantification in the culture medium of both biofilms was lower compared to the buffer (*p* < 0.05) and the culture medium in the absence of bacteria, the CHX system was able to display antibiofilm effects until 96 h for the *S. mutans* biofilms (*p* < 0.05) and 72 h for the *P. gingivalis* biofilms (*p* < 0.05). (4) Conclusions: The experimental formulation is able to extend chlorhexidine effects, even in challenging conditions such as in the presence of bacteria, allowing the in vitro control of cariogenic biofilms for 4 days and periodontopathogenic biofilms for 3 days.

## 1. Introduction

Biofilm is the major etiological factor for the development of dental caries [1] and is intimately associated with the advancing lesions of periodontitis [2]. Although clinical evidence shows that oral mechanical hygiene is fundamental to prevent and control these oral diseases, most individuals are not able to achieve sufficient biofilm control by brushing [3,4]. Thus, the complementary use of chemotherapeutic agents such as chlorhexidine has been investigated as a way to overcome the deficiencies of mechanical oral hygiene habits [5].

Chlorhexidine, also known as chlorhexidine gluconate (CHX), is one of the most commonly prescribed antiseptic agents in dentistry and it is considered a gold standard for dental caries and periodontitis control [3,6]. A notable feature of CHX is that it adheres to tooth and mucosal surfaces, which allows a prolonged antimicrobial activity inside the oral cavity [7]. However, the mouth environment is also complex and the constant presence of saliva may cause much of the drug to be washed off and lost, requiring repeated dosing to obtain a therapeutic dose [8]. In addition, the microenvironment produced by the biofilm could reduce the activity of potent agents such as CHX by preventing antimicrobial diffusion through the deeper layers of the matrix [9]. Based on these issues, the incorporation of CHX into a formulation capable of extending its antimicrobial effect might allow a wide spacing of dosages, optimizing the pharmacologic feature of this molecule and improving biofilm control.

Antimicrobials require time to exert their effects and semi-solid systems can extend release of drugs, offering multiple benefits such as an increment in drug retention times at the site of application and reduction of side effects [10]. Although attractive, these properties can be compromised by the presence of oral bacterial enzymes which interact with the formulation components and interfere with drug release [8]. Moreover, microorganism metabolism can produce high levels of acids [11] or organic bases [12], causing pH oscillations that may also compromise drug release. These observations prompted us to develop a formulation containing CHX, quantifying it in buffer solution or dissolution medium in the presence/absence of bacteria, with the aim of analyzing the potential of this semi-solid formulation facing some challenges presented by oral cavity. We also evaluated the antimicrobial effects produced by this formulation using two pathogenic biofilm models, one representative of dental caries and the other for periodontitis. We believe that this formulation could improve the pharmacologic features of CHX and extend its antibacterial effect.

## 2. Materials and Methods

### 2.1. Materials

Chlorhexidine digluconate solution (CHX, 20% in H_2_O) was obtained from Fagron (Saint Paul, MN, USA) and hydroxypropyl methylcellulose (HPMC, Methocel K100LV) was obtained from Colorcon (Harleysville, PA, USA). Chitosan (medium molecular weight; deacetylation degree of 75–85%), hemin, menadione, sodium chloride, sodium phosphate monobasic, sodium phosphate dibasic, magnesium sulfate and diethylamine were purchased from Sigma-Aldrich (Sant Louis, MO, USA). Acetic acid and formic acid were obtained from Labsynth (Diadema, SP, Brazil), methanol was obtained from J.T. Baker (Phillipsburg, NJ, USA), potassium phosphate dibasic was purchased from Vetec (Rio de Janeiro, RJ, Brazil), sucrose was purchased from Merck (Darmstadt, HE, Germany) and glucose was purchased from Fluka (St. Gallen, Switzerland). Tryptone, Brain Heart Infusion (BHI), Brain Heart Infusion agar (BHI-agar) and blood agar base were supplied from Oxoid (Basingstoke, Hampshire, UK). The yeast extract and the anaerobe container systems with the indicator were purchased from BD Diagnostics (Sparks, MD, USA). Microscopic glass slides (76 × 26 × 1.2 mm) were obtained from Knittelglaser (Varrentrappstr, Braunschweig, Germany). Ultrapure water from Milli-Q water system was used to prepare the aqueous solutions. All other chemicals used in this study were of analytical grade.

### 2.2. Chlorhexidine-Based Chitosan Semi-Solid System Preparation

Dispersions of chitosan at 4% (*w*/*v*) were prepared by dissolving chitosan in 0.1 M acetic acid solution with overnight magnetic stirring at room temperature to promote the complete dispersion of chitosan. After stabilization of the chitosan dispersion, HPMC and lyophilized CHX were incorporated. CHX lyophilization was necessary to achieve the drug concentration required for the formulation proposal. The pH was adjusted to a final value of 7.0 and the formulation stability was checked after 24 h at room temperature. The final composition of the semi-solid formulation was 82% chitosan, 8% HPMC and 10% CHX. The formulation was stored in syringes and sterilized by X-rays (130 Gy, 160 kV, 25 mA, 0.3 mm Copper filter) for 30 min (Rad Source’s RS 2000 Biological Research Irradiator, Shanghai Medicilon, Shanghai, China), according to Ré et al. [13].

### 2.3. Biofilm Growth

*Streptococcus mutans* UA 159 biofilms were formed according to Koo et al. [14] with slight modifications. Briefly, the *S. mutans* biofilms were grown on glass microscope slides immersed in buffered tryptone yeast-extract broth containing 1% sucrose, for 6 days, at 37 °C with 5% CO_2_. On the third day, the glass slides with biofilms were placed in tubes containing the culture medium and exposed to treatments [13]. Two independent experiments were performed in triplicate (*n* = 3). The culture medium was replaced daily.

*Porphyromonas gingivalis* W83 biofilms were formed according to Papaioannou et al. [15], the main modification being the use of glass slides as the surface. *P. gingivalis* was grown on glass slides coated with human saliva (approved on 25 June 2015 by Research Ethics Committee of School of Pharmaceutical Sciences of Ribeirão Preto, identification code 376) for 4 days, at 37 °C and under anaerobic conditions (jars with anaerobe container system). The slides were individually positioned in 50-mL tubes containing 45 mL of the bacterial inoculum (1.5 × 10^8^ bacteria/mL) with BHI broth (supplemented with 5 μg/mL hemin and 1 μg/mL menadione) and exposed to treatments (*n* = 3). The culture medium was replaced daily.

### 2.4. Treatments

A vertical thin strip (515 ± 10 mg) of the formulation with CHX or a control formulation without the drug (92% chitosan and 8% HPMC as vehicle control, VC) was individually positioned in 50-mL tubes containing 45 mL of fresh culture medium for each bacterium. A biofilm group without exposure to any formulation was included as negative control (NC). For *S. mutans*, the biofilms and culture medium were collected at 24, 48, 72, and 96 h after exposure to the CHX formulation. For *P. gingivalis*, the biofilm and culture medium were collected at 24, 48, and 72 h after exposure to the CHX formulation. Immediately prior to CHX quantification (item 2.5), the pH of the culture medium was measured using a digital pHmeter (PG2000 model, Gehaka, São Paulo, SP, Brazil) while the biofilms were analyzed for bacterial viability (item 2.6). Preliminary tests showed that the formulation was able to remain adhered to the tube for 7 days (data not shown), preserving characteristics such as the shape and color during all experimental periods.

### 2.5. Chlorhexidine Quantification

At the end of each experimental period, the culture medium from the *S. mutans* or *P. gingivalis* biofilms was used to evaluate the quantity of CHX released from the formulation (presence of bacteria). Samples were prepared according to Ré et al. [13]. Briefly, the whole culture medium (45 mL) was centrifuged, filtered and methanol (1:2) was added to the filtrate before it was stored at −20 °C. After 24 h, the samples were defrosted, centrifuged and the supernatants were filtered. This last centrifugation step was repeated three times. The filtrates were analyzed by High Performance Liquid Chromatography (HPLC) on a chromatograph (Prominence; Shimadzu, Kyoto, Japan), with the diode array detector (DAD-UV, SPDM20A model) set at 254 nm, using a C-18 column (250 × 4.6 mm; 5.0-μm particle size, Shim-pack VP-ODS) and C-18 security guard. The mobile phase contained methanol (65%) and ultrapure water with formic acid (35%; pH 3.5) and 0.4% of diethylamine (*v*/*v*), in isocratic mode and under a flow rate of 0.8 mL/min at 40 °C (*n* = 3).

In order to verify if the mechanism governing CHX release was affected by the presence of bacteria, a vertical thin strip (515 ± 10 mg) of the formulation with CHX was individually positioned in 50-mL tubes containing 45 mL of: (i) culture medium only, pH 7.2, at 37 °C (absence of bacteria) or (ii) 30 mM sodium phosphate buffer, pH 6.8, at 37 °C (buffer solution). At the end of the experimental period (24, 48, 72, and 96 h after exposure to the CHX formulation), each dissolution medium was withdrawn and quantified in relation to its CHX content. The results were expressed as the percentage of CHX quantified from the formulation (calculated by comparing the initial amount of the CHX in the formulation vs. the amount of CHX quantified in the culture medium at all times) and the CHX concentration (percentage, *w*/*v*) in different conditions.

### 2.6. Bacterial Viability

After 24, 48, 72 and 96 h, the *S. mutans* biofilms were washed three times in 0.9% NaCl to remove any non-adherent bacteria and carefully scraped to collect at least 10 mg of wet biofilm. For *P. gingivalis*, the biofilm collections were performed after 24, 48 and 72 h and the biofilms were washed and removed with a swab. Each biofilm was transferred into microcentrifuge tubes containing 1 mL of 0.9% NaCl and sonicated to improve homogenization [16]. The biofilm suspensions were diluted serially and inoculated [17] in BHI-agar or BHI-agar blood plates (supplemented with 5 μg/mL hemin and 1 μg/mL menadione) for *S. mutans* and *P. gingivalis* growth, respectively. The colony-forming units (CFU) were counted and the results were expressed in CFU/mg of wet biofilm for *S. mutans* and CFU/mm^2^ for *P. gingivalis*.

### 2.7. Statistics

The data were analyzed by SAS System software (SAS Institute Inc., release 9.3, 2012, Cary, NC, USA). The assumption of equality of variances and normal distribution of errors was checked and the Tukey–Kramer (bacterial viability and acidogenicity of *S. mutans* and CHX quantification in *P. gingivalis* culture medium) and Tukey test (bacterial viability of *P. gingivalis*, CHX quantification in *S. mutans* culture medium) were used to compare the means of significant effects with the significance level fixed at 5%.

## 3. Results

### 3.1. pH in Culture Medium

The pH of *S. mutans* biofilms exposed to the CHX formulation (Table 1) was higher and significantly different among other groups (*p* < 0.05). Also, in CHX group pH at 48 and 72 h increased significantly in relation to 24 h (*p* < 0.05). However, there was no difference between the pH at 72 and 96 h (*p* > 0.05). The biofilms exposed to the NC and VC rapidly lowered the pH at all times (24, 48, 72 and 96 h). No differences were observed between these two groups, which indicates that the vehicle control does not affect the acid production by *S. mutans* (*p* > 0.05).

The pH obtained for the *P. gingivalis* biofilms was 7.0 ± 0.0 for all studied groups (NC, VC and CHX) and did not change over time (24, 48 and 72 h).

### 3.2. Chlorhexidine Quantification

Table 2 shows the percentage of CHX quantified in three different dissolution media: buffer solution, culture medium in the absence of bacteria (tryptone yeast-extract broth) and culture medium from *S. mutans* biofilm growth, along with the respective CHX concentrations.

The highest and lowest values for CHX quantification and concentrations were observed in the buffer and the culture medium from the *S. mutans* biofilms at all times, respectively (*p* < 0.05). The culture medium in the absence of bacteria had a higher amount of CHX compared to the culture medium from the biofilms at all times (*p* < 0.05).

Table 3 shows the results for CHX percentage quantified in the buffer solution, culture medium in the absence of bacteria (BHI broth with hemin and menadione) and culture medium from *P. gingivalis* biofilms, along with the respective CHX concentrations.

There was a significant reduction in the CHX concentration percentage of the dissolution media (buffer, culture medium in the absence of bacteria and culture medium from biofilm) from 24, 48 to 72 h (*p* > 0.05). The highest and lowest values for CHX quantification were observed in the buffer and the culture medium from the *P. gingivalis* biofilms at all times, respectively (*p* < 0.05). In addition, the CHX percentage in the culture medium from the *P. gingivalis* biofilms was about 1.5 times lower than the CHX quantified in the culture medium in the absence of bacteria at all times (*p* < 0.05).

### 3.3. Biofilm Analyses

**Figure 1:**
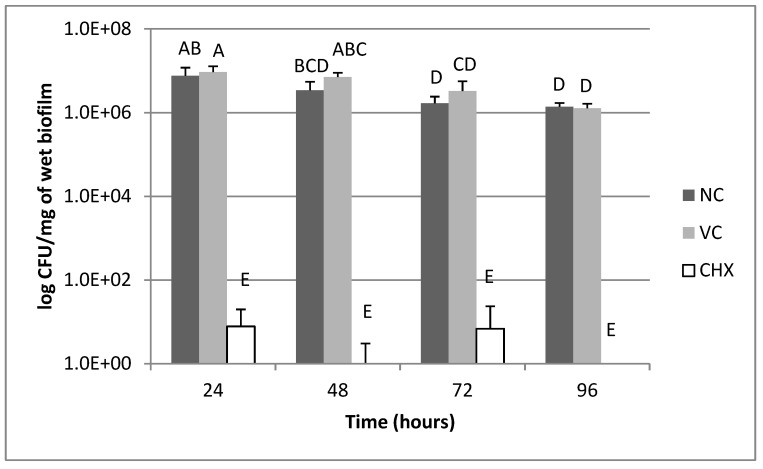
Bacterial viability of *S. mutans* biofilms according to treatments. NC= negative control (without exposure to formulation); VC= vehicle control; CHX= chlorhexidine-based chitosan formulation. Data expressed as mean ± standard deviation in logarithmic scale (*n* = 6). Values that do not share the same letter (A, B, C, D, E) are significantly different from each other; Shapiro–Wilk test followed by Tukey–Kramer post-hoc test, *p* < 0.05.

Figure 1 shows the *S. mutans* biofilms results for bacterial viability according to the treatments. Biofilm exposure to the chlorhexidine-based chitosan formulation (CHX) resulted in lower bacterial viability compared to the negative control (NC) and vehicle control (VC) at all times (*p* < 0.05). For the NC and VC groups, there was a decrease in bacterial viability at 72 and 96 h compared to 24 h (*p* < 0.05).

**Figure 2:**
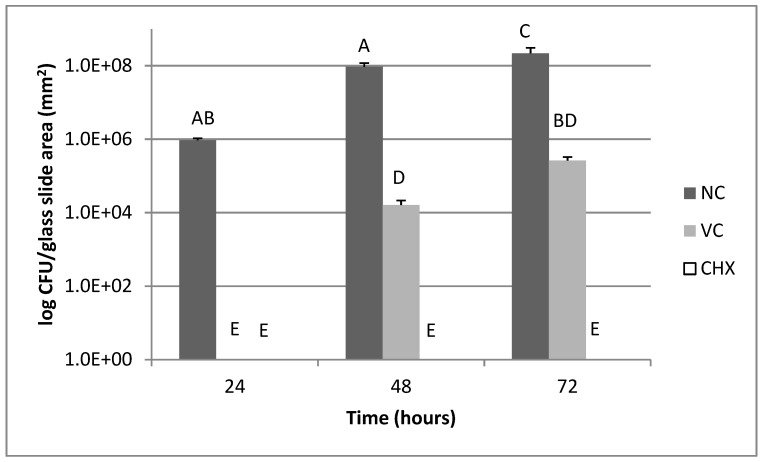
Bacterial viability of *P. gingivalis* biofilms according to treatments. NC= negative control (without exposure to formulation); VC= vehicle control; CHX= chlorhexidine-based chitosan formulation. Data expressed as mean ± standard deviation in logarithmic scale (*n* = 6). Values that do not share the same letter (A, B, C, D, E) are significantly different from each other; Shapiro–Wilk test followed by Tukey–Kramer post-hoc test, *p* < 0.05.

Regarding bacterial viability of *P. gingivalis* biofilms (Figure 2), the lowest bacterial count was found after treatment with the CHX group at all times (*p* < 0.05) but did not differ from VC at 24 h. The VC group was different from the NC group for all times (*p* > 0.05). The NC group achieved the highest bacterial viability in 72 h compared to 24 and 48 h (*p* < 0.05).

## 4. Discussion

Chlorhexidine (CHX) is an antimicrobial agent commonly used as a mouthwash and presents a high residence time compared to other oral antimicrobials [18]. The possibility of improving this property, using a semi-solid formulation, may have advantages in the control of pathogenic biofilms. Our data support the view that the experimental formulation can extend the effect of CHX through a high initial drug release with strong bactericidal activity during and after contact with biofilms, which prolonged the effect of CHX.

The bactericidal effects were seen on the elevated pH of the *S. mutans* biofilms after exposure to the CHX formulation compared to the controls at all times studied (Table 1). The ability of CHX to reduce the acidogenicity of the *S. mutans* biofilms has been established [6,19]. However, the CHX concentrations that produce this effect are usually higher than those found in the present study. For example, Lee et al. [6] and Ccahuana-Vasquez and Cury [19] showed a decrease in acidogenicity after exposure to 0.04% and 0.012% chlorhexidine solutions, respectively. In our case, concentrations ranging from 0.002 to 0.008% were enough to promote the same inhibition on the *S. mutans* biofilms (Table 2). The incorporation of CHX in a semi-solid formulation probably continually exposed the biofilms to the antimicrobial agent, which may produce a different effect than occasional exposure to the chlorhexidine solution performed in the cited studies. In fact, the continued exposure of the *S. mutans* biofilms to these lower concentrations also had an inhibitory effect on the bacterial viability data (Figure 1). The results of CHX quantification showed that the lowest concentration obtained at 96 h (0.002%) was able to exert bactericidal effects even in the presence of a structured biofilm.

Although pH changes in the *P. gingivalis* biofilms were not observed, the CHX quantified had an effect on the inhibition of biofilm formation for all times. In contrast to the *S. mutans* biofilms, the *P. gingivalis* biofilms were susceptible to the vehicle control, showing a decrease in bacterial viability, especially in 24 h (Figure 2). Chitosan probably contributed by enhancing the initial antimicrobial effects since its biological activities are well established in literature [20] and its presence as a vehicle could improve formulation design as well as formulation bioadhesive properties. In our study, chitosan was used to increase the formulation residence time and the results reinforce its bioadhesive property: CHX was released slowly and detected until 96 h for the *S. mutans* biofilms and until 72 h for *P. gingivalis* biofilms. Thus, the use of chitosan as a polymeric carrier may potentiate the biological effects of the CHX system.

The quantification of CHX in the culture medium from biofilm growth was lower compared to the other two media at each time point. However, the composition of those media is different between each other. Microbiological culture medium contains substrates essential for bacterial development such as salts (which dissociate into their component inorganic ions in aqueous solution), peptone, proteins and carbohydrates. The complexity of culture medium increases in the presence of a biofilm, since a variety of inflammatory mediators and tissue-destructive molecules—including metalloproteinases and metabolic signatures associated with host–bacterial interactions, trace metals, metabolites, toxin neutralizers, biochemicals, proteins, glycoproteins and lipids—could be expressed by bacteria. All those components could influence the ionic strength of the dissolution medium and influence drug release from the formulation. However, the CHX quantified in the culture medium may not represent the total amount of drug released from the formulation. It is possible that CHX binds to specific biofilm components or be degraded by bacterial enzymes, which should be investigated in future studies.

Considering that patients with imperfect oral hygiene present a pathogenic biofilm, it is important to consider bacteria presence in oral formulation design, simulating the ionic composition of biological fluids as closely as possible. Thus, in the present work, we exposed some of those factors to an experimental formulation using pathogenic bacterium, evaluating such delivery systems using doses able to disorganize complex biofilms. Since our results suggest that the experimental formulation is able to improve the effects of CHX, the next step is to characterize our experimental formulation, making a rational design in order to develop a product to be used in dentistry. This highlights the relevance of combining microbiological models as part of drug dissolution assays, especially for formulations in the early stages of development such as the one presented in this study.

## 5. Conclusions

The experimental formulation proposed is able to release chlorhexidine even in challenging conditions such as the presence of bacteria, allowing the in vitro control of cariogenic biofilms for 4 days and periodontopathogenic biofilms for 3 days. Further characterization of this semi-solid system will contribute to select formulation strategies for enhancing the clinical performance of chlorhexidine in dentistry.

## Figures and Tables

**Table 1:** Effect of the chlorhexidine-based chitosan formulation (CHX) on the pH of the *S. mutans* biofilms at 24, 48, 72 and 96 h.

Treatment Groups	Time after First Treatment Exposure (h)			
	24	48	72	96
NC	3.9 ± 0.0 ^A^	3.9 ± 0.0 ^A^	4.0 ± 0.0 ^A^	3.9 ± 0.1 ^A^
VC	4.0 ± 0.1 ^A^	3.9 ± 0.0 ^A^	3.9 ± 0.0 ^A^	3.9 ± 0.1 ^A^
CHX	5.8 ± 0.1 ^B^	6.7 ± 0.0 ^C^	6.8 ± 0.1 ^D^	6.8 ± 0.0 ^D^

NC= negative control (without exposure to formulation); VC= vehicle control; CHX= chlorhexidine-based chitosan formulation. Data expressed as mean ± standard deviation (*n* = 6). Values that do not share the same letter (A, B, C, D) are significantly different from each other; Shapiro–Wilk test followed by Tukey–Kramer post-hoc test, *p* < 0.05.

**Table 2:** Percentage of chlorhexidine (CHX) quantified and the final CHX concentration (in percentage) in the buffer, culture medium in the absence of bacteria and the culture medium from *S. mutans* biofilm growth at 24, 48, 72 and 96 h.

Time of Exposure to Treatments (h)	Dissolution Medium					
	Buffer Solution		Culture Medium in the Absence of Bacteria		Culture Medium from *S. mutans* Biofilm Growth	
	% of CHX Quantified	[CHX] in %	% of CHX Quantified	[CHX] in %	% of CHX Quantified	[CHX] in %
24	22.8 ± 0.9 ^A^	0.025 ± 0.0	13.6 ± 0.6 ^B^	0.016 ± 0.0	6.9 ± 1.2 ^EF^	0.008 ± 0.0
48	11.9 ± 0.8 ^BC^	0.013 ± 0.0	10.6 ± 1.4 ^CD^	0.012 ± 0.0	3.4 ± 0.2 ^GH^	0.004 ± 0.0
72	9.0 ± 0.2 ^DE^	0.010 ± 0.0	7.8 ± 0.3 ^E^	0.011 ± 0.1	1.9 ± 0.1 ^H^	0.002 ± 0.0
96	8.9 ± 1.3 ^DE^	0.010 ± 0.0	5.1 ± 0.5 ^FG^	0.006 ± 0.0	1.8 ± 0.2 ^H^	0.002 ± 0.0

CHX: chlorhexidine-based chitosan formulation. Data were analyzed intra-group and expressed as mean ± standard deviation (*n* = 3). Values that do not share the same letter (A to H) are significantly different from each other; Shapiro–Wilk test followed by Tukey or Tukey–Kramer post-hoc test, *p* < 0.05.

**Table 3:** Percentage of chlorhexidine (CHX) quantified and the final CHX concentration (in percentage) in the buffer, culture medium in the absence of bacteria and the culture medium from *P. gingivalis* biofilm growth at 24, 48 and 72 h.

Time of Exposure to Treatments (h)	Dissolution Medium					
	Buffer Solution		Culture Medium in the Absence of Bacteria		Culture Medium from *P. gingivalis* Biofilm Growth	
	% of CHX Quantified	[CHX] in %	% of CHX Quantified	[CHX] in %	% of CHX Quantified	[CHX] in %
24	22.8 ± 0.9 ^A^	0.025 ± 0.0	17.6 ± 0.3 ^B^	0.020 ± 0.0	11.3 ± 0.3 ^C^	0.013 ± 0.0
48	11.9 ± 0.8 ^C^	0.013 ± 0.0	10.8 ± 0.2 ^C^	0.012 ± 0.0	7.2 ± 0.1 ^EF^	0.008 ± 0.0
72	9.0 ± 0.2 ^D^	0.010 ± 0.0	6.5 ± 0.1 ^F^	0.007 ± 0.0	4.1 ± 0.4 ^G^	0.005 ± 0.0

CHX: chlorhexidine-based chitosan formulation. Data were analyzed intra-group and expressed as mean ± standard deviation (*n* = 3). Values that do not share the same letter (A to G) are significantly different from each other; Shapiro–Wilk test followed by Tukey or Tukey–Kramer post-hoc test, *p* < 0.05.
